# Frailty and length of stay in older adults with blunt injury in a national multicentre prospective cohort study

**DOI:** 10.1371/journal.pone.0250803

**Published:** 2021-04-30

**Authors:** Timothy Xin Zhong Tan, Nivedita V. Nadkarni, Wei Chong Chua, Lynette Ma Loo, Philip Tsau Choong Iau, Arron Seng Hock Ang, Jerry Tiong Thye Goo, Kim Chai Chan, Rahul Malhotra, Marcus Eng Hock Ong, David Bruce Matchar, Dennis Chuen Chai Seow, Hai V. Nguyen, Yee Sien Ng, Angelique Chan, Ting-Hway Wong

**Affiliations:** 1 Emergency Medicine Residency Program, SingHealth Services, Singapore, Singapore; 2 Centre for Quantitative Medicine, Duke-NUS Graduate Medical School, Singapore, Singapore; 3 Trauma Service, Tan Tock Seng Hospital, Singapore, Singapore; 4 Department of General Surgery, National University Hospital, Singapore, Singapore; 5 Accident & Emergency, Changi General Hospital, Singapore, Singapore; 6 Department of General Surgery, Khoo Teck Puat Hospital, Singapore, Singapore; 7 Emergency Medicine Department, Ng Teng Fong General Hospital, Singapore, Singapore; 8 Health Services and Systems Research, Duke-NUS Graduate Medical School, Singapore, Singapore; 9 Department of Emergency Medicine, Singapore General Hospital, Singapore, Singapore; 10 Department of Geriatric Medicine, Singapore General Hospital, Singapore, Singapore; 11 School of Pharmacy, Memorial University of Newfoundland, Canada, St. John’s, NL, Canada; 12 Department of Rehabilitation Medicine, Singapore General Hospital, Singapore, Singapore; 13 Centre for Ageing Research and Education, Duke-NUS Graduate Medical School, Singapore, Singapore; 14 Department of General Surgery, Singapore General Hospital, Singapore, Singapore; John Hunter Hospital and University of Newcastle, AUSTRALIA

## Abstract

**Background:**

Patients suffering moderate or severe injury after low falls have higher readmission and long-term mortality rates compared to patients injured by high-velocity mechanisms such as motor vehicle accidents. We hypothesize that this is due to higher pre-injury frailty in low-fall patients, and present baseline patient and frailty demographics of a prospective cohort of moderate and severely injured older patients. Our second hypothesis was that frailty was associated with longer length of stay (LOS) at index admission.

**Methods:**

This is a prospective, nation-wide, multi-center cohort study of Singaporean residents aged ≥55 years admitted for ≥48 hours after blunt injury with an injury severity score or new injury severity score ≥10, or an Organ Injury Scale ≥3, in public hospitals from 2016–2018. Demographics, mechanism of injury and frailty were recorded and analysed by Chi-square, or Kruskal-Wallis as appropriate.

**Results:**

218 participants met criteria and survived the index admission. Low fall patients had the highest proportion of frailty (44, 27.3%), followed by higher level fallers (3, 21.4%) and motor vehicle accidents (1, 2.3%) (p < .01). Injury severity, extreme age, and surgery were independently associated with longer LOS. Frail patients were paradoxically noted to have shorter LOS (p < .05).

**Conclusion:**

Patients sustaining moderate or severe injury after low falls are more likely to be frail compared to patients injured after higher-velocity mechanisms. However, this did not translate into longer adjusted LOS in hospital at index admission.

## Introduction

As populations age and life expectancies increase, trauma in the elderly has more than doubled within the last decade [1]. This is a common cause of death [2], with low falls being the most common mechanism of injury (MOI), followed by motor vehicle accidents (MVAs) and high falls [1, 2]. Although there are varying definitions of low fall such as <2 meters and patient height [2], a lower definition of <0.5 meters has been proposed [3–5], in keeping with video-capture studies demonstrating that most falls in the elderly happen from the seated or standing position [3]. In previous studies, we found that low falls were associated with higher readmission and mortality rates compared to higher-velocity mechanisms such as MVAs [4, 5]. This is postulated to be because low falls are more likely to occur in frail patients. The complexity of examining outcomes in the frail elderly probably also explains the finding that low fall patients are more likely to die of causes seemingly unrelated to the initial trauma [4, 5].

Age, gender, comorbidities, and injury severity (Injury Severity Score (ISS), Revised Trauma Score (RTS)) [4] do not account for the poorer outcomes in some older trauma patients. Frailty [6, 7], characterized by reduced physiologic reserve [8], is associated with fall risk [9], readmissions [4, 8, 9], in-hospital complications [6], prolonged hospital stays [10], decreased quality of life [6, 7], increased healthcare costs and overall mortality [2, 7]. We hypothesize that older patients sustaining moderate or severe injury after low falls are more likely to be frail at baseline compared to older patients injured after higher-velocity MOI. In this study, we present the baseline patient demographics and frailty measures of a prospective multi-center nationwide cohort study of older blunt trauma patients who survived the index admission and examine the association between frailty and length of stay (LOS) in hospital at index admission.

## Methods

### Background

Singapore is a rapidly aging Asian city with a life expectancy of 83.1 years and a population of 5.6 million [5], of whom 24.6% are 55 years and older, compared to 17.3% worldwide [11].

### Study design

Prospective nationwide, multi-center cohort study.

### Patient and public involvement

This research was done without patient involvement. Patients were not invited to comment on the study design and were not consulted to develop patient relevant outcomes or interpret the results. Patients were not invited to contribute to the writing or editing of this document for readability or accuracy.

### Data source and data collection

Singapore residents aged ≥55 years admitted for ≥48 hours after blunt injury with an injury severity score (ISS) or new injury severity score (NISS) ≥10, or an Organ Injury Scale (OIS) ≥ three at any government hospital from Mar 2016 to Jul 2018 were screened for participation in the study. As this was designed as a one-year prospective cohort study, patients who did not survive the index admission were excluded, and patients or caregivers were only approached when patients were in the general ward (i.e. not in intensive care or high dependency). The SingHealth Institutional Review Board granted ethical approval for this study and all research was performed in accordance with the relevant guidelines and regulations. Informed consent to participate was obtained in writing from the patients and/or caregivers after confirming with the primary attending physicians that it was appropriate to approach the patients and/or caregivers. For patients unable to participate due to cognitive impairment (either pre-injury or due to head injury), their caregivers were approached for the caregiver arm of the study. Patients were not approached if: the primary attending physician did not provide consent for the study team to approach the patient (patients not expected to survive the index admission would have been included in this group), or if the patient could not give consent and there was no caregiver. ISS data were drawn from Singapore National Trauma Registry (NTR) offices at the respective government hospital study sites.

Demographics (age, gender, race, housing type), injury characteristics (MOI, ISS, NISS, polytrauma [12]), admitting discipline, LOS, surgical intervention, and discharge destination were recorded at the index admission. MOI was categorized into low falls (<0.5m, including falls from standing height), high falls (≥0.5m), and MVAs; polytrauma was defined as having Abbreviated Injury Scale (AIS) scores of ≥ three in at least two body regions [12–14].

Pre-injury functional status (Barthel Index) [15], Modified Fried’s Criteria (MFC) [16] and Modified Frailty Index (mFI-11) [8, 17] were calculated for each patient. Frailty by MFC was defined as the presence of three of more of the following: (1) unintentional weight loss (≥5 kilograms in the last year), (2) slowness (slow walking speed, based on the question, “Do you find it difficult to walk 200 to 300 meters (one bus stop to another)?”) [18], (3) exhaustion (present if answered “yes” to at least one of the two questions from the Center for Epidemiological Studies–Depression Scale “I felt that everything I did was an effort” / “I could not “get going””) [16], (4) weakness (grip strength) [11] and (5) low physical activity [16]. To calculate pre-injury MFC scores, weight loss, slowness and exhaustion were assessed via a survey questionnaire [18]. Grip strength was measured with a dynamometer at time of recruitment [11] and physical activity assessed via the Global Physical Activity Questionnaire (GPAQ), both validated locally [19]. The mFI-11, an 11-point frailty index validated to correlate with both morbidity and mortality [17], was calculated from functional status (Barthel Index of ≥80) and comorbidities. Although both MFC and mFI-11 are associated with poor outcomes in database-based studies [5], MFC has been demonstrated to have a stronger association with poor outcomes such as postoperative morbidity, LOS and unplanned readmissions at one year in our local population [20]. Other scales were not examined to reduce participant fatigue, as patients were recruited during hospital admission.

### Statistical analysis

Data was reported as the mean (SD) or median (IQR) for continuous variables, and frequency (%) for categorical variables. Chi-square or Fisher’s exact was used to compare categorical variables by MOI, while Kruskal-Wallis was used to compare categorical and continuous variables across the MOI categories where appropriate. For the secondary analysis, univariate analysis was performed to examine the association between demographic and clinical (injury severity, mechanism of injury) variables, and LOS of more than seven days. In the multivariable model, both statistically and clinically significant variables were included. Threshold for statistical significance was set at p < .05. The MFC definition of frailty was used as the primary indicator of frailty, as it appears to have a stronger association with poorer outcomes [16, 21]. Sensitivity analysis used MFI-11 instead of MFC as a frailty measure. All statistical analyses were carried out using STATA 15.1(Texas).

## Results

**Fig 1:**
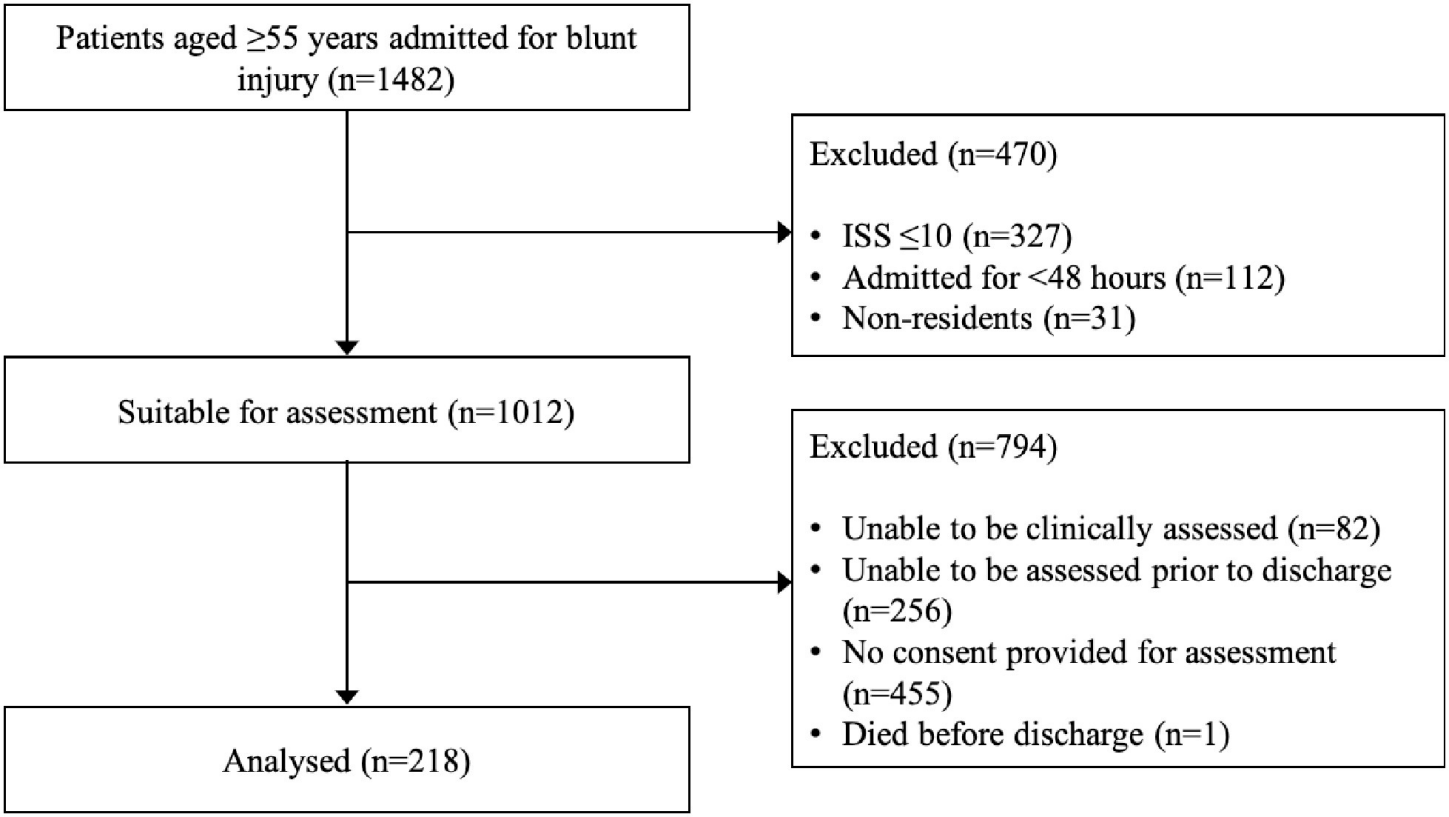
Participant recruitment flowchart.

Out of 1482 patients aged 55 years and older screened based on injury severity to be entered into the NTR, 31 patients were non-residents, 112 were admitted for <48 hours, 327 had ISS ≤10, one of the recruited patients died before discharge, 82 could not be clinically assessed, 256 could not be reviewed prior to discharge, and consent was not provided for 455 patients (Fig 1). Of the remaining 218 patients, 70 (32.1%) answered by proxy (patients cognitively impaired). Mean age was 74 years old (range 56–100 years old), 57.3% were male, 57.7% were married, and 22.0% had pre-injury frailty (Table 1).

**Table 1:** Demographics, frailty, injury severity and discharge destination by mechanism of injury.

	Total	Low Fall (<0.5m)	High Fall (≥0.5m)	Motor Vehicle Accident	p-value
**Demographics**					
Study Population (%)	218 (100.0)	161 (73.9)	14 (6.4)	43 (19.7)	**-**
Male (%)	125 (57.3)	80 (49.7)	8 (57.1)	37 (86.0)	**< .001**^†^
Female (%)	93 (42.7)	81 (50.3)	6 (42.9)	6 (14.0)	**-**
Age, years (mean/SD)	74.1 (10.8)	76.8 (10.2)	72.1 (10.8)	64.6 (7.2)	**< .001**^‡^
**Function and Frailty**					
Barthel Index (median/IQR)	100 (90–100)	100 (90–100)	100 (99–100)	100 (100–100)	**< .001**^‡^
Functionally dependent at baseline (Barthel Index <80)	25 (11.5)	24 (14.9)	1 (7.1)	0 (0.0)	**.02**^†^
Modified Fried’s Criteria (MFC)^§^ (median/IQR)	1 (1–2)	2 (1–3)	1 (0–2)	1 (1–2)	**< .01**^‡^
Non-Frail (%)	30 (13.8)	19 (11.8)	4 (28.6)	7 (16.3)	**< .01**^†^
Pre-Frail (%)	140 (64.2)	98 (60.9)	7 (50.0)	35 (81.4)	-
Frail (%)	48 (22.0)	44 (27.3)	3 (21.4)	1 (2.3)	-
Modified Frailty Index (mFI-11) (median/IQR)	2 (1–3)	2 (1–3)	2 (1–2)	1 (1–3)	.41^†^
Non-Frail (%)	19 (8.7)	14 (8.7)	0 (0.0)	5 (11.6)	
Pre-Frail (%)	127 (58.3)	90 (55.9)	11 (78.6)	26 (60.5)	
Frail (%)	72 (33.0)	57 (35.4)	3 (21.4)	12 (27.9)	
**Injury Severity**					
Injury Severity Score (ISS) (median/IQR)	14 (10–18)	13 (10–17)	15 (10–26)	14 (10–22)	**.04**^‡^
10–15	133 (61.0)	104 (64.6)	7 (50.0)	22 (51.2)	
16–24	58 (26.6)	40 (24.8)	2 (14.3)	16 (37.2)	
≥25	27 (12.4)	17 (10.6)	5 (35.7)	5 (11.6)	
New Injury Severity Score (NISS) (median/IQR)	18 (13–27)	17 (11–25)	22 (13–33)	22 (17–27)	**.04**^‡^
Polytrauma (%)	26 (11.9)	10 (6.2)	3 (21.4)	13 (30.2)	**< .01**^‡^
**Management**					
ICU Admission (%)	33 (15.1)	22 (13.7)	4 (28.6)	7 (16.3)	.33^‡^
Length of Stay (median/IQR)	12 (6–23)	12 (7–22)	12 (6–33)	12 (6–25)	.65^‡^
Underwent Surgery (%)	87 (39.9)	56 (34.8)	6 (42.9)	25 (58.1)	**.01**^‡^
Admitting Discipline (%)					
Medicine (Geriatrics, Rehab etc.)	35 (16.0)	28 (17.4)	3 (21.4)	4 (9.3)	**< .001**^†^
General Surgery/Trauma	39 (17.9)	14 (8.7)	4 (28.6)	21 (48.8)	-
Orthopaedics	47 (21.6)	32 (19.9)	3 (21.4)	12 (27.9)	-
Neurosurgery	97 (44.5)	87 (54.0)	4 (28.6)	6 (14.0)	-
Discharge Destination (%)					
Own Residence	117 (53.6)	82 (50.9)	6 (42.9)	29 (67.4)	.25^‡^
Inpatient Rehabilitation / Step-down Care	94 (43.1)	72 (44.7)	8 (57.1)	14 (32.6)	-
Long-Term-Care/Nursing Home	7 (3.2)	7 (4.4)	0 (0.0)	0 (0.0)	-

^†^ Chi-square

^‡^ Kruskal-Wallis

^§^ Non-frail, pre-frail and frail have been defined by Fried et al. as the presence of 0, 1–2, and ≥3 of the following 5 criteria respectively: unintentional weight loss, slowness, exhaustion, weakness, and low physical activity (Fried).

The most common mechanism of injury was low fall (n = 161, 73.9%), followed by MVA (n = 43, 19.7%), and high fall (n = 14, 6.4%), out of which six fell from a height of 0.5-1m, four from a height of 1-2m and four from a height ≥2m. MVA patients were younger (64.6 years old, SD 7.2) with the ages of high fall patients lying between the low fall and MVA patients (72.1 years old, SD 10.8).

Median ISS was 14 (IQR 10–18) and 26 (11.9%) patients met criteria for anatomical polytrauma (AIS score of three or more in two or more ISS body regions). In keeping with higher likelihood of multi-system injury, MVA patients had the highest proportion of anatomical polytrauma (30.2%), followed by high falls (21.4%) and low falls (11.9%) (p < .01). 33% were admitted to the ICU, 39.9% eventually underwent some form of surgical intervention, and overall median hospital LOS was 12 days (IQR 6–23).

Low fall patients had the highest proportion of MFC frailty (44, 27.3%), and functional dependence at baseline (14.9%, p = .02), followed by higher level fallers (frail 3, 21.4%; functionally dependent 1, 7.1%) and MVA patients (frail 1, 2.3%; functional dependence 0, 0%) (p < .01, 02). The commonest admission discipline was neurosurgery (n = 97, 44.5%), followed by orthopaedics (n = 47, 21.6%), with low fall (n = 87, 54.0%) patients mostly admitted to neurosurgery and MVA patients (n = 21, 48.8%) to general surgery/trauma (p < .001). There was no significant difference by mechanism of injury between the proportions of patients admitted to intensive care, LOS, and discharge destination. A lower proportion of frail patients (13, 27%) underwent surgery compared to pre-frail or non-frail patients (74, 43.5%) (p = .04) (additional patient demographics are detailed in S1 Table).

On univariate analysis, patient ISS, undergoing surgery, and anatomical polytrauma were associated with LOS of more than 7 days (ISS 16–24: OR 2.74, 95%CI 1.30–5.77, p = .01, ISS≥25: OR 3.69, 95%CI 1.21–11.28, p = .02, undergoing surgery: OR 6.89, 95%CI 3.18–14.89, p < .001, polytrauma: OR 3.18 95%CI 1.29–7.84, p = .01) (Table 2). Frailty was not associated with LOS (p = .07) on univariate analysis.

**Table 2:** Multivariable regression for length of stay.

	Univariate		Multivariate	
	Odds Ratio (95% CI)	p-value	Odds Ratio (95% CI)	p-value
Gender				
Male (female ref)	0.80 (0.44–1.43)	.44	0.92 (0.45–1.87)	.82
Age (years)				
55–64 (ref)	1	-	1	-
65–74	0.94 (0.44–2.01)	.88	1.36 (0.53–3.53)	.52
75–84	1.51 (0.66–3.46)	.33	2.51 (0.86–7.36)	.09
≥85	1.45 (0.59–3.54)	.42	3.03 (0.97–9.43)	.06
Frailty (MFC)				
Non-Frail/Pre-Frail (ref)	1	-	1	-
Frail	0.51 (0.24–1.07)	.07	0.43 (0.19–0.99)	**.05**
Mechanism of Injury				
Low Fall (<0.5m) (ref)	1	-	1	-
High Fall (≥0.5m)	1.09 (0.33–3.66)	.88	1.24 (0.30–5.11)	.77
Motor Vehicle Accident	0.91 (0.44–1.86)	.79	0.73 (0.27–1.93)	.52
Injury Severity Score (ISS)				
10–15 (ref)	1	-	1	-
16–24	2.74 (1.30–5.77)	**.01**	3.22 (1.36–7.64)	**.01**
≥25	3.69 (1.21–11.28)	**.02**	1.55 (0.42–5.74)	.51
Polytrauma (AIS ≥3 in ≥2 body regions)	3.18 (1.29–7.84)	**.01**	1.65 (0.59–4.59)	.34
Underwent Surgery (%)	6.89 (3.18–14.89)	**< .001**	8.61 (3.59–20.66)	**< .001**

On multivariable analysis, in addition to the abovementioned statistically significant variables, clinically significant variables based on hypotheses for this study (frailty, mechanism of injury) and requirements recommended in the literature (age, gender, injury severity) [22, 23] were included (Table 2). Injury severity (ISS 16–24 compared to ISS 10–15, (OR 3.16, 95%CI 1.33–7.48, p = .01)), extreme age (age 85 years or more, compared to age 55–64, (OR 3.12, 95%CI 1.01–9.66, p = .05) and surgical intervention (OR 8.68, 95%CI 3.63–20.78, p < .001) were significantly associated with increased LOS. Paradoxically, frail patients had a shorter LOS compared to non-frail patients (OR 0.44, 95%CI 0.19–0.99, p < .05). Sensitivity analyses incorporating interactions between surgery and frailty, and surgery and ISS showed no significant interactions between these terms, and did not change our findings. Sensitivity analysis replacing MFC with MFI-11 did not change our findings.

## Discussion

As populations age, the medical and surgical care of the frail patient deserves greater attention [3, 7, 8]. Frailty is a known risk factor for adverse outcomes in surgical and trauma populations [4, 6, 7]. Our study focuses on comparing frailty across different mechanisms of injury for older blunt trauma patients. Low fall patients were shown to have the highest proportion of MFC frailty and functional dependence at baseline, followed by “intermediate-frailty” higher-level-fall patients, with MVA patients having the lowest proportion of frailty. Indeed, low fallers were noted in previous studies to be twice as likely to die within a year of injury compared to other MOI, followed by higher-level fallers and MVA patients [4, 5].

Although patients suffering a low fall were more likely to be admitted to non-surgical disciplines than patients admitted after high-velocity injury, many low fall patients still get admitted to surgical departments, such as neurosurgery, orthopaedics or general surgery. This reflects the real-world need for identification of high-risk populations and inter-disciplinary care in planning healthcare and the prioritization of intervention for the older injured patient [1, 4].

However, the low fallers in our study were noted to have slightly lower ISS, NISS, polytrauma and surgical intervention rates compared to other MOI. After adjusting for age, gender, injury severity, MOI and surgery, frail patients had a shorter LOS compared to non-frail patients. We postulate that the shorter LOS is partly attributable to such patients being unfit for surgery, surgeon preference for lower-risk conservative management, or the pattern of injuries that were less likely to need surgery despite adjusting for overall injury severity. This explanation is supported by the lower surgical intervention rates in such patients (p = .01). Several studies have demonstrated that frail patients are also more likely to already have support systems such as home safety interventions in place pre-injury, justified by the higher functional dependence observed at baseline (14.9%, p = .02). Such systems facilitate early discharge and could possibly account for the shorter LOS observed during index admission [1, 10]. This finding also highlights that outcomes for such frail, older, moderate to severe trauma patients at index admission may appear falsely reassuring, as registry-based studies have demonstrated that such patients have worse post-discharge outcomes (readmission and death) [4, 6, 7]. Studies that only consider inpatient or 30-day outcomes may miss the longer-term poorer outcomes related to frailty, reported in previous registry-based studies that formed the rationale for this prospective study [4, 5].

### Limitations

This study has the following limitations: Firstly, there is under-representation of patients who are not cognitively fit to give consent and yet do not have a caregiver—patients expected to be of higher risk than the general population. However, the proportion of these common blunt mechanisms of injury in our study was fairly similar to the Singapore national trauma registry-based study [4], and we hope to have minimized this participation bias in the study, by including caregivers in our study. One-third of responses also relied on a proxy, limiting subjective aspects (e.g. isolation) from being assessed in this subset.

Pre-injury weakness is difficult to assess due to a lack of information on preinjury function or frailty factors [5]. Grip strength assessed post-injury was therefore used as a surrogate measure which is in turn affected by injury and subsequent disability and may have resulted in an overestimate of frailty [4]. Future studies might consider using other frailty scores for example, Clinical Frailty Scale, which has since been studied in emergency surgical patients, or the 15-Variable Trauma-Specific Frailty Index [7, 24, 25].

Finally, public ambulances convey all major trauma to public hospitals, but patients presenting to private hospitals would not be represented in our study. However, this has a minor effect on our data as public ambulance usage is high [22].

## Conclusion

Older patients sustaining moderate and severe injury due to low falls have been shown to suffer higher morbidity and mortality than other higher-velocity mechanisms of injury. This study presents the baseline patient demographics and frailty of older moderately and severely injured blunt trauma patients in Singapore. We demonstrate that the low fall mechanism of injury is associated with a higher proportion of baseline frailty, yet frailty was associated with a reduced length of stay at index admission.

## Supporting information

S1 Table Supplementary demographics by mechanism of injury. (DOCX)
