# Supplementary material for: Frailty and length of stay in older adults with blunt injury in a national multicentre prospective cohort study
Source: PLoS One. 2021 Apr 30;16(4):e0250803. doi: 10.1371/journal.pone.0250803 (PMC8087011; doi:10.1371/journal.pone.0250803)
Supplement: S1 Table — (DOCX) [file pone.0250803.s001.docx]

**S1 Table. Supplementary Demographics by Mechanism of Injury.**

|  | **Total** | **Low Fall (<0.5m)** | **High Fall (≥0.5m)** | **Motor Vehicle Accident** | **p-value** |
| --- | --- | --- | --- | --- | --- |
| **Demographics** | | | | | |
| Study Population (%) | 218 (100.0) | 161 (73.9) | 14 (6.4) | 43 (19.7) | **-** |
| Answered by Proxy (%) | 70 (32.1) | 50 (31.1) | 7 (50.0) | 13 (30.2) | **.33** |
| Ethnicity (%) | | | | | |
| Chinese | 184 (84.4) | 140 (87.0) | 14 (100.0) | 30 (70.0) | **<.01**^‡^ |
| Malay | 23 (10.6) | 11 (6.8) | 0 (0.0) | 12 (27.9) | **-** |
| Indian | 11 (5.0) | 10 (6.2) | 0 (0.0) | 1 (2.3) | **-** |
| Marital Status (%) | | | | | |
| Married | 126 (57.7) | 81 (50.3) | 10 (71.4) | 35 (81.4) | **<.001**^‡^ |
| Unmarried (Single/Widowed/ Divorced) | 92 (42.3) | 80 (49.7) | 4 (28.6) | 8 (18.6) | **-** |
| Level of Education (%) | | | | | |
| Primary / Not Educated | 116 (53.2) | 95 (59.0) | 4 (28.6) | 17 (39.5) | **<.01**^‡^ |
| Secondary | 78 (35.8) | 51 (31.7) | 7 (50.0) | 20 (46.5) | - |
| Tertiary | 24 (11.0) | 15 (9.3) | 3 (21.4) | 6 (14.0) | - |
| Employment Status (%) | | | | | |
| Full-time | 54 (24.8) | 25 (15.5) | 3 (21.4) | 26 (60.5) | **<.001**^‡^ |
| Part-time | 17 (7.8) | 10 (22.8) | 0 (0.0) | 7 (16.2) | - |
| Retired/Homemaker | 147 (67.4) | 126 (61.7) | 11 (78.6) | 10 (23.3) | - |
| Housing Type (%) | | | | | |
| 1-2 room government housing | 25 (11.5) | 19 (11.8) | 1 (7.1) | 5 (11.6) | .34^‡^ |
| 3-4 room government housing | 115 (52.8) | 88 (54.7) | 6 (42.9) | 21 (48.8) | - |
| >4 room government housing | 47 (21.5) | 33 (20.5) | 2 (14.3) | 12 (27.9) | - |
| Private housing | 31 (14.2) | 21 (13.0) | 5 (35.7) | 5 (11.6) | - |

^‡^ Kruskal-Wallis
